# 

**Published:** 1881-04-20

**Authors:** 


### TABLE OF CONTENTS.

ORIGINAL AND SELECTED ARTICLES.

Conversations upon the Physical and Mental Hygiene of Girlhood, by T. S. Powell, M.D, Atlanta, 121. Gonorrhœa, by A. F. Durham, M.D., of Georgia, 129. Progressive Locomotor Ataxy, by T. B. Greenley, M.D., of Kentucky, 131. Double Pneumonia, by E. P. Townsend, M.D., 133. Chronic Nasal Catarrh, 136.

ABSTRACTS AND GLEANINGS.

Treatment of Ozæna, 137. Inhalation of Sulphuric Ether, 138. Etiology of Vertigo, 139. Indigestion and Heartburn, 140. Tongue as a Diagnostic Point in Diseases, 141. Phlyctenular Conjunctivitis, 142. For Catarrh of the Nasal Passage; Sulphate of Hyoscyamin as a Mydriatic, 143. Iodine in Diphtheria; Alstonia Constricta; Trichina Spiralis, 144. Potassium Bromide in Infantile Diarrhœas; Ovariotomy; Artificial Vaccine, 145. Abortive and Curative Treatment of Small Pox; Treatment of Nævus; Treatment of Carbuncle, 146. Tripolith a Substitute for Plaster of Paris; A New Narcotic; Despotism in Lunatic Asylums, 147. Diphtheria; Organisms in Abdominal Typhus; Ergot, 148. Hemorrhoids; A Powerful Antiseptic; Notes on Diphtheria; Delirium in Acute Rheumatism from Salicylate of Soda, 149. A New Method of Inhalation; To Terminate the Chloroform Narcosis; For Asthma; Resection of Rectum, 150.

SCIENTIFIC ITEMS.

Recent Progress in Photography, 151. Man in America; Germs of Disease in Water, 152.

PRACTICAL NOTES AND FORMULÆ.

Raw Meat Juice in Cases of Inanition from Irritable Stomach; For Anemia of Chlorosis; Simple Continued Fever, 153. Sore Nipples; Pruritus of Pregnancy; Cystitis; For Frost-Bite; Antidote for Carbolic Acid, 154. For Spermatorrhœa; Remedy for Corns, 155. Ipecac in Dyspepsia; Copaiva in Sciatica; Gleet, 156.

EDITORIAL AND MISCELLANEOUS.

Editorial Notices; American Medical Association, 157. Book Notices, 158. Special Notices, 160.

